# Global Map of Specialized Metabolites Encoded in Prokaryotic Plasmids

**DOI:** 10.1128/spectrum.01523-23

**Published:** 2023-06-13

**Authors:** Zaki Saati-Santamaría

**Affiliations:** a Departamento de Microbiología y Genética, Universidad de Salamanca, Salamanca, Spain; b Institute for Agribiotechnology Research (CIALE), Villamayor, Salamanca, Spain; c Institute of Microbiology of the Czech Academy of Sciences, Prague, Czech Republic; Institut Pasteur

**Keywords:** megaplasmids, microbial ecology, plasmids, secondary metabolism

## Abstract

Plasmids are the main mobile elements responsible for horizontal gene transfer (HGT) in microorganisms. These replicons extend the metabolic spectrum of their host cells by carrying functional genes. However, it is still unknown to what extent plasmids carry biosynthetic gene clusters (BGCs) related to the production of secondary or specialized metabolites (SMs). Here, we analyzed 9,183 microbial plasmids to unveil their potential to produce SMs, finding a large diversity of cryptic BGCs in a few varieties of prokaryotic host taxa. Some of these plasmids harbored 15 or more BGCs, and many others were exclusively dedicated to mobilizing BGCs. We found an occurrence pattern of BGCs within groups of homologous plasmids shared by a common taxon, mainly in host-associated microbes (e.g., *Rhizobiales*, *Enterobacteriaceae* members). Our results add to the knowledge of the ecological functions and potential industrial uses of plasmids and shed light on the dynamics and evolution of SMs in prokaryotes.

**IMPORTANCE** Plasmids are mobile DNA elements that can be shared among microbial cells, and they are useful for bringing to fruition some microbial ecological traits. However, it is not known to what extent plasmids harbor genes related to the production of specialized/secondary metabolites (SMs). In microbes, these metabolites are frequently useful for defense purposes, signaling, etc. In addition, these molecules usually have biotechnological and clinical applications. Here, we analyzed the content, dynamics, and evolution of genes related to the production of SMs in >9,000 microbial plasmids. Our results confirm that some plasmids act as a reservoir of SMs. We also found that some families of biosynthetic gene clusters are exclusively present in some groups of plasmids shared among closely related microbes. Host-associated bacteria (e.g., plant and human microbes) harbor the majority of specialized metabolites encoded in plasmids. These results provide new knowledge about microbial ecological traits and might enable the discovery of novel metabolites.

## INTRODUCTION

Microbes are a valuable source of metabolites useful in medicine, agriculture, and industry due to their bioactivities, such as antimicrobial, antiviral, antitumoral, and siderophore activity (1–3). These natural products are mainly produced through secondary metabolism, and the genes responsible for their biosynthesis are usually clustered within biosynthetic gene clusters (BGCs). Nonetheless, researchers in this area are beginning to refer to many of these metabolites as “specialized” molecules rather than secondary metabolites (4–6); this trend is changing the anthropocentric view of microbes as cell factories toward a more ecological view in which their natural products are considered relevant molecules for interacting with the environment and the microbiome (e.g., signaling, niche defense) (7–11). Due to both the ecological relevance and industrial applications of microbial natural products, much research is being conducted to unveil the biosynthetic potential of microbes and describe their evolution through genome analyses (2, 6, 12–14). Indeed, this research is on the increase due to the large availability of microbial genomes worldwide (2, 15).

Beyond the use of chromosomal sequences, those of microbial plasmids are also becoming broadly available (16–18). The increasing use of long-read sequencing facilitates the assembly of these replicons (19, 20). Plasmids are key drivers of horizontal gene transfer (HGT) and are transferred by conjugation, transduction, transformation, and vesiduction (21). Recently, Redondo-Salvo et al. (17) categorized over 10,000 plasmids into plasmid taxonomic units (PTUs)—clusters of plasmids with high average nucleotide identity—and assigned them a host range (species, genus, family, order, class, or phylum), if any. The authors found that more than 60% of plasmids can be transferred among strains differentiated at taxonomic levels higher than species. Most plasmids carry genes that impact the ecological adaptation and environmental interaction of microbes (21). In the best-known cases, those referred to as antimicrobial resistance and virulence genes spread through plasmids (22, 23). Beyond this, many other cases reflect the importance of accessory genes within plasmids; for instance, rhizobia carry large plasmids encoding relevant functions for their symbiotic relationship with legumes, such as nitrogen fixation and nodulation factors (21, 24). Additionally, the family *Rhodobacteraceae* gained its ability to undergo anoxygenic photosynthesis due to genes carried on plasmids (25). Similarly, a few BGCs have been described from plasmids, mainly hosted by actinobacteria (26–28), but to date, the secondary and specialized metabolites encoded in microbial plasmids have never been addressed at a global scale, and our knowledge of the BGC diversity in prokaryotic plasmids remains limited.

The presence of BGCs within plasmids deserves a special attention in the field of natural product research. First, plasmids represent a niche for drug discovery that has not been investigated in depth, which means that the possibility of isolating and identifying a known microbial metabolite encoded in a plasmid is low. Second, the facility of manipulating plasmids in laboratory experiments makes research on these BGCs easier. In addition, such research will have an important impact on the fields of microbial ecology and genome evolution, by addressing the study of functions that can be both horizontally and vertically transmitted and that can also affect chromosome configurations (21, 27).

Here, we analyzed the BGCs carried on more than 9,000 microbial plasmids, mainly from prokaryotic hosts, to provide new insight into the general conjunction of specialized metabolites (SMs). We focused on the distribution of BGCs within PTUs and among plasmid hosts, finding large gene cluster clans (GCC)—clusters of closely related gene cluster families (GCFs)—exclusively present in ecologically related taxa (e.g., host-associated microbes), and we also found that some plasmids are devoted to encoding natural products. These results impact our understanding of ecological functions shared among microbes and may be useful for prioritizing efforts in natural product research.

## RESULTS

### Some prokaryotic taxa harbor plasmids with a broad repertoire of BGCs.

**FIG 1:**
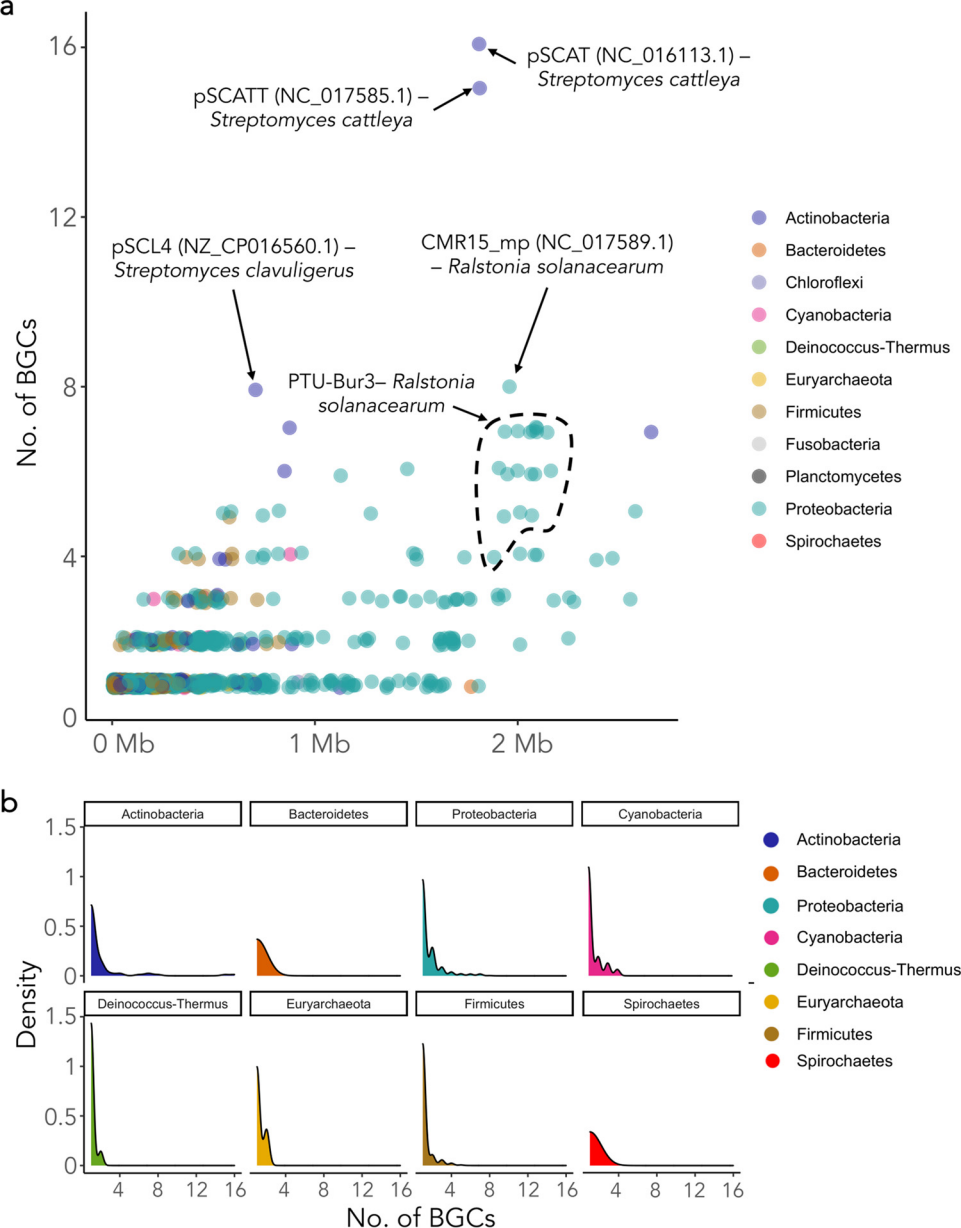
Density of BGCs within plasmids from diverse host cell taxa. (a) Representation of the number of BGCs harbored within prokaryotic plasmids (*y* axis) and the plasmid size (*x* axis). Some plasmids with a high BGC content and diversity are highlighted (GenBank accession numbers are shown in parentheses). (b) Density of BGCs in plasmids from different phyla. Phyla with no BGC-carrying plasmids are not displayed.

We searched for genes related to the biosynthesis of secondary/SMs in 9,183 plasmids hosted by 23 microbial phyla (see File S1 in the supplemental material). We found 1,560 BGCs carried on 1,029 plasmids from 11 different prokaryotic phyla (File S2). A total of 271 of these plasmids harbored 2 or more BGCs (Fig. 1; File S1). The distribution of BGCs among host microbes was highly dissimilar; for instance, 58% of the analyzed *Rhizobiales* plasmids contained BGCs (499/1,560 BGCs) (File S2). Similarly, the orders *Pseudonocardiales* (48%), *Legionellales* (34%), and *Streptomycetales* (31%) also contained a high proportion of plasmids with BGCs (File S2). In contrast, plasmids from some taxa, such as the phylum *Spirochaetes* (2 BGCs out of 1,077 plasmids) and the genus Pseudomonas (2 BGCs out of 109 plasmids), rarely carried BGCs.

**FIG 2:**
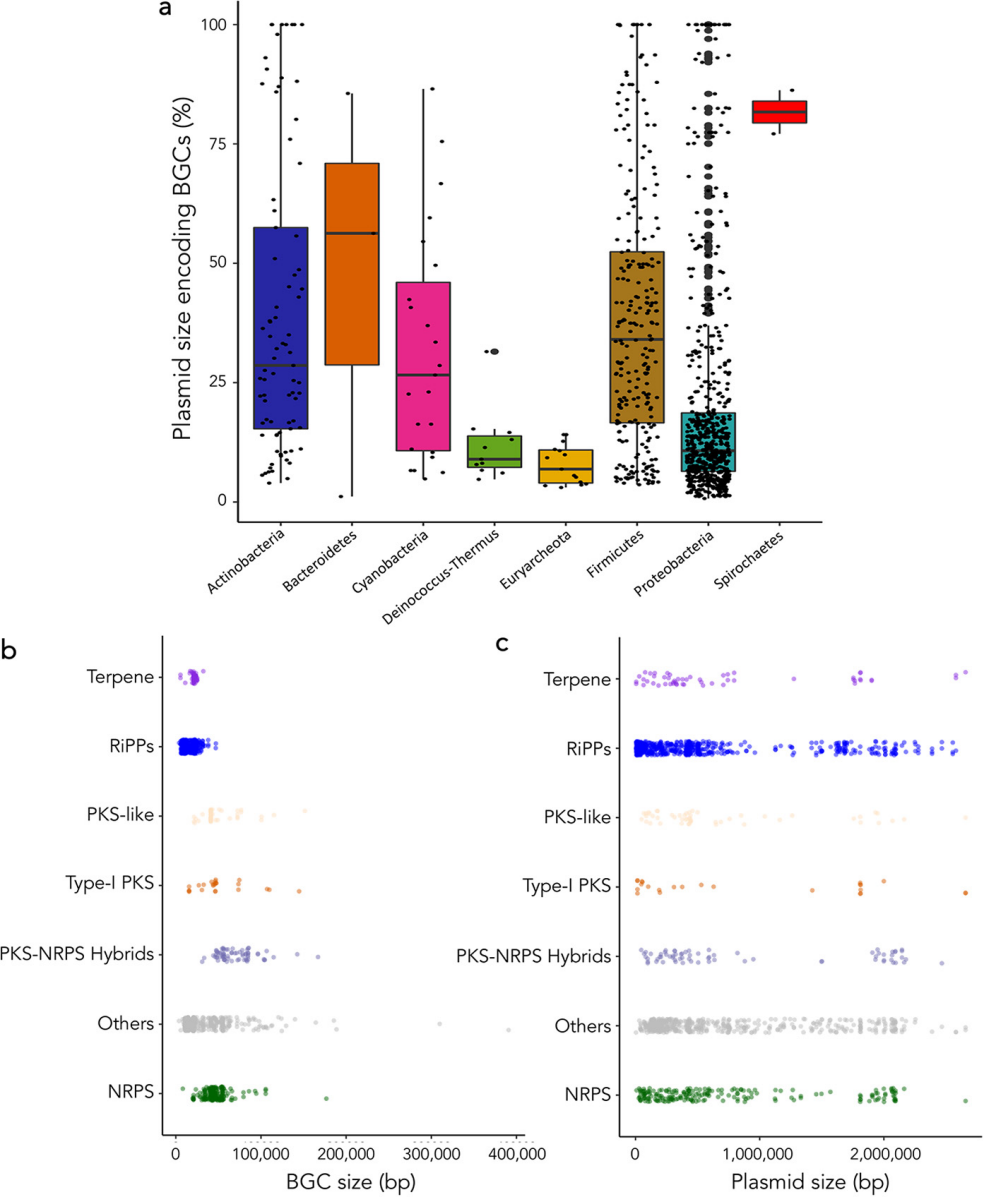
BGC content and coverage within prokaryotic plasmids. The length of some plasmids is as long as the sum of the lengths of their BGCs, while many other plasmids dedicate most of their size for other purposes (a). These results are not an effect of the BGC type or size (b). In addition, the occurrence of diverse BGC types is not related to plasmid size (c). Note: the BGC region sizes might be overestimated due to *in silico* prediction biases.

The proportion of the plasmid covered by BGCs differed between microbial phyla (Fig. 2a); for example, *Euryarchaeota* plasmids that carried BGCs dedicated >80% of their sequences to non-SM-related genes. However, interestingly, we found some plasmids encoding a large number of SMs, such as 2 Streptomyces cattleya plasmids containing 16 and 15 BGCs (pSCAT and pSCATT, respectively) (Fig. 1). Moreover, some plasmids were dedicated to producing BGCs: we found 37 whose total length was covered by a BGC, while 168 plasmids dedicated >50% of their sizes to producing SMs (Fig. 2a).

Plasmid size and BGC content were positively correlated, mostly in actinobacteria (*R*^2^ = 0.59; *P* = 9.93e−86) (Fig. 1; Fig. S1). Nonetheless, this association was not linked to the type of BGC carried. Large BGCs (e.g., polyketide synthase-nonribosomal peptide synthetase [PKS-NRPS] hybrids) and small BGCs (e.g., terpenes) (Fig. 2b) were detected in plasmids of very divergent sizes (Fig. 2c).

### Diversity and novelty of biosynthetic gene clusters in plasmids.

**FIG 3:**
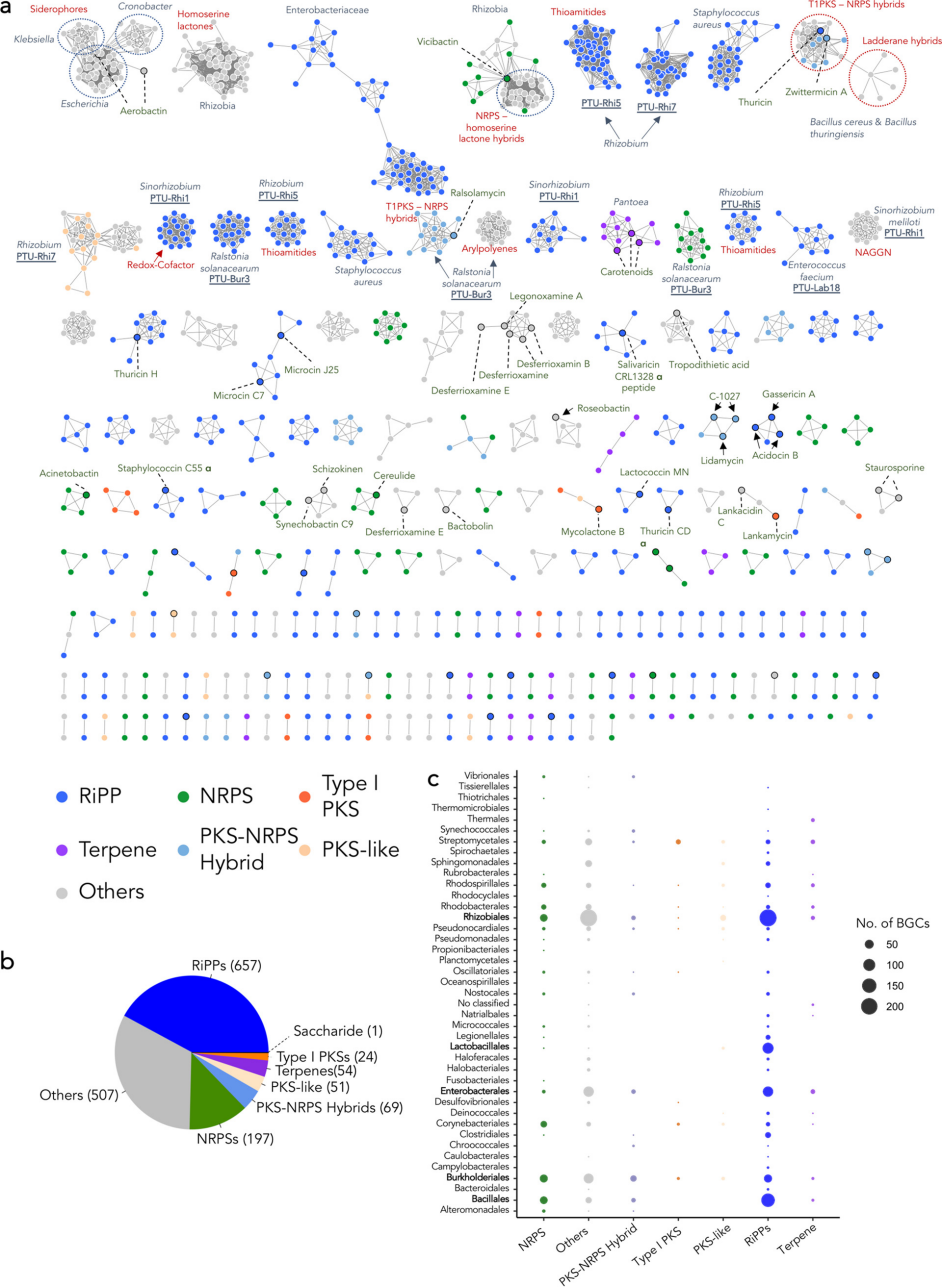
Diversity of BGCs within plasmids. (a) Sequence similarity network (SSN) built based on BGC distances. Each dot represents a BGC, colored according to the BGC type, and those sharing high similarity are connected with gray lines. Gene cluster families (GCFs) and/or GCF clades are grouped within independent clusters. The network includes previously described BGCs (from the MIBiG database) closely related to those found in the plasmid data set, indicated with a black, dotted circle. The MIBiG-BGCs found in the largest clusters of BGCs are labeled with their encoded product. GCFs present exclusively in plasmids from certain host taxa (species, genus, family, or order) are accompanied by the taxon name, including those solely found in PTUs. Red text specify certain types of large BGC clusters classified as RiPPs or others. (b) Representation of the total number of BGCs of each type found in plasmids, including singletons. (c) Number of BGCs of each type in the plasmids of each of the host cell orders in this study.

We analyzed the similarity of the predicted BGCs in plasmids using BiG-SCAPE. This analysis produced 806 gene cluster families (GCFs) and 23 GCCs (Fig. 3a). Of them, 571 GCFs represented unique BGCs (singletons) that were distant from any other BGCs found in this analysis. We also compared our detected BGCs to those available in the MIBiG database to assess their potential novelty or relatedness to known SMs. While a few large clusters were similar to some already described BGCs (e.g., those producing aerobactin, zwittermicin A, thuricin, vicibactin), the majority appeared to encode novel metabolites (Fig. 3a).

The most abundant type of BGC within the plasmids encompassed ribosomally synthesized and posttranslationally modified peptides (RiPPs) (Fig. 3b), mainly carried on *Rhizobiales*, *Bacillales*, *Enterobacterales*, and *Lactobacillales* plasmids (Fig. 3c). PKSs were mainly found in *Streptomycetales* plasmids (Fig. 3c).

### Horizontal gene transfer of SMs through plasmids among closely related host bacteria.

**FIG 4:**
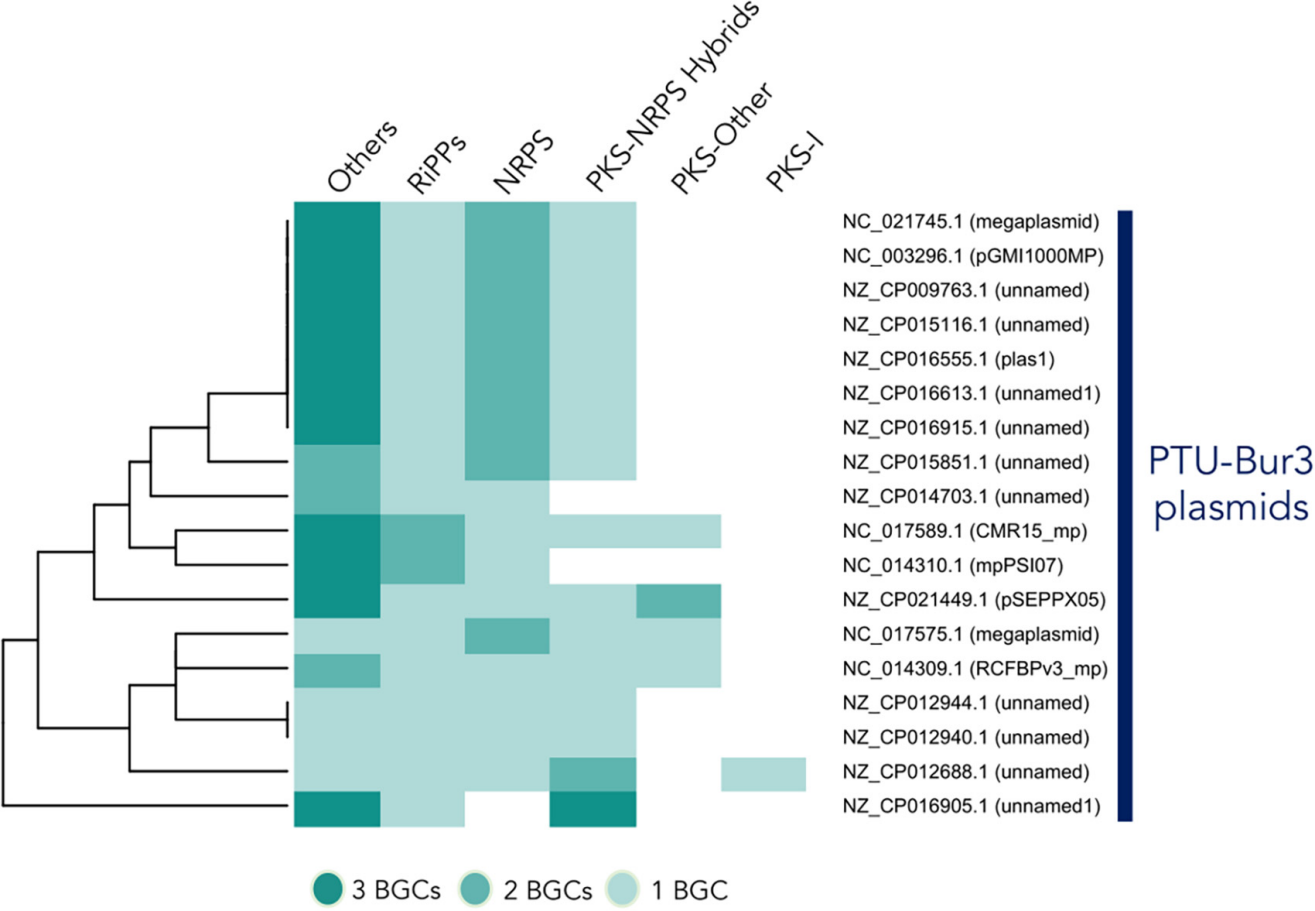
Biosynthetic potential of Ralstonia solanacearum PTU-Bur3 plasmids (shown with their GenBank accession numbers). The number of BGCs from each type in Ralstonia solanacearum PTU-Bur3 plasmids is shown, and the plasmids are ordered by their patterns of occurrence and abundance.

Equally to microbial genomes, plasmids can also be clustered within taxonomic units (PTUs) based on their similarity (17). However, a link between the SM profile or BGC similarities and PTU or plasmid host taxonomy has never been demonstrated. Here, we show that many closely related BGCs are exclusively present within some PTUs (Fig. 3a). For instance, the rhizobium PTU-Rhi1 (*Sinorhizobium*), PTU-Rhi5 (*Rhizobium*), PTU-Rhi7 (*Rhizobium*), and Enterococcus faecium PTU-Lab18 contained GCFs exclusive to each of these PTUs (Fig. 3a). Ralstonia solanacearum PTU-Bur3 represents a group of plasmids with a large number of BGCs (*n* = 4 to 8) (Fig. 1 and 4), many of which are highly similar within the PTU (Fig. 3a). Similarly, we found some large clusters of BGCs shared among PTUs but exclusive for some host species (e.g., Staphylococcus aureus, Bacillus cereus, Bacillus thuringiensis), families (e.g., *Enterobacteriaceae*), or orders (e.g., *Rhizobiales*) (Fig. 3a).

**FIG 5:**
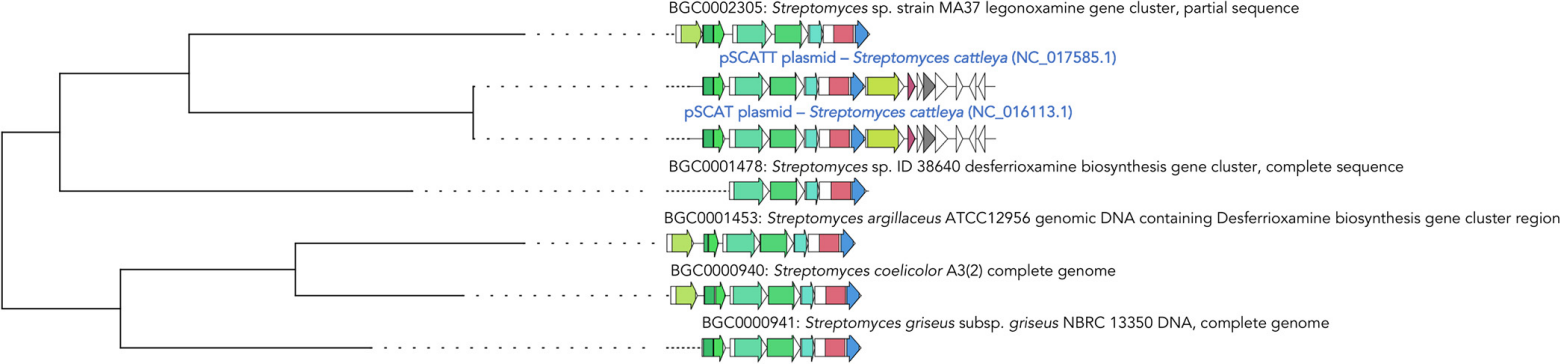
Desferrioxamine gene cluster family. The phylogeny of the GCF was built based on desferrioxamine clusters found in *S. cattleya* plasmids (blue text), including several homologs described in chromosomal units (black text).

Plasmids are key drivers of microbial HGT, as they might be easily shared among cells, which means that their genes are also shared among different microbes. We found highly similar BGCs present in different genera, which suggests that these genes may have been horizontally transferred or vertically transmitted before the evolutionary divergence of those taxa (Fig. 3a). Furthermore, plasmid genes can be acquired by a microbial chromosome and persist therein. Here, we found a pair of BGCs in *Streptomyces cattleya* plasmids that shared the same genetic structure as some desferrioxamine-related BGCs in chromosomal sequences (Fig. 5). Nonetheless, we found no other cases like this one among those BGCs closely related to known BGCs.

## DISCUSSION

Plasmids are important mobile elements for microbial evolution, and their genes can facilitate ecological adaptation for microorganisms or even be relevant in host-microbe interactions (21, 29). SMs are molecules that, beyond their relevance for such ecological interactions, also have great biotechnological importance (5, 30). However, their presence in plasmids has not been investigated in depth. Here, we unveil a large diversity of BGCs encoding some known and many likely unknown SMs. These findings provide strong evidence of how this type of biosynthetic gene can be easily shared among microbial cells. Overall, these BGCs have no known homologs within the chromosomes of any strains, and those already described have been found in plasmids. Thus, it is possible that the presence of SMs in plasmids may derive from ancestral events in which these replicons gained genes and/or suffered BGC reorganizations, rather than the exclusion of these BGCs directly from chromosomes, with the unique exception of desferrioxamine clusters. Nonetheless, it is not known to what extent the presence of these BCGs in plasmids (and not in chromosomes) would be beneficial or detrimental, in evolutionary terms, for bacteria. More research data are needed to ascertain their origin, since the chromosomal exclusion may reflect also a recent BGC evolution that has not been integrated into chromosomes yet due to lack of evolutionary time or adaptation pressure.

We found that some large GCFs with highly similar BGCs were exclusively present within rhizobial plasmids (Fig. 3a). Rhizobia are microbes that establish symbiosis with plants in roots and, specifically, in N_2_-fixing nodules (31, 32). We found a large clan of BGCs related to the production of *N*-acyl homoserine lactones (AHLs) in rhizobial plasmids. These quorum-sensing molecules are known to regulate relevant functions for N_2_-fixing symbiosis and can even be perceived by the host plant, impacting this interdomain interaction (33, 34). We also found a cluster of BGCs likely related to *N*-acetylglutaminylglutamine amide (NAGGN) production within a Sinorhizobium meliloti PTU (PTU-Rhi1). NAGGN is a dipeptide that enhances plant adaptation to high osmolarity environments (35–37). Therefore, its bacterial production is beneficial for plant survival in extreme environments, which represents a plant growth-promoting trait relevant to biofertilizer design. Similarly, siderophores are Fe-scavenging molecules known to be useful for competing in the rhizosphere environment and for facilitating iron acquisition by plants (38–40). Vicibactin is one such rhizobial siderophore that is produced by an NRPS system (41). Several BGCs similar to those involved in vicibactin production were found in rhizobial plasmids. Indeed, this metabolite has already been linked with Rhizobium etli plasmids (42), but our findings extend this biosynthetic potential to plasmids from diverse *Rhizobiales* families. Moreover, we found a clan of PKS-like BGCs with no resemblance to any known BGC. Nonetheless, its maintenance within *Rhizobium* PTU-Rhi7 may be justified by some yet-unknown functions relevant for plant-bacteria interactions. Overall, our findings expand the ecological relevance of rhizobial plasmids to fulfil more useful plant-beneficial traits.

Additionally, several clans of thioamitide-related BGCs were found in *Rhizobium* plasmids (PTU-Rhi5 and PTU-Rhi7). Thioamitides are antitumor molecules mainly isolated from *Streptomyces* strains (43, 44). Due to the lack of similarity to previously described BGCs, research on the cryptic DNA material encoding thioamide biosynthesis in rhizobial plasmids may lead to profitable drug discoveries. However, its biological role in natural ecosystems or microbiomes remains unknown. Further research is needed to understand why these gene clusters are conserved within the genus *Rhizobium*.

In contrast, plasmids can also facilitate bacterial pathogenicity. One such case is illustrated by the Ralstonia solanacearum species complex, in which several disease-related effectors, among other pathogenic traits, have been found to be carried in plasmids (45). Here, we report that a cluster of similar plasmids from R. solanacearum (PTU-Bur3) is specifically enriched in SM-producing BGCs (Fig. 1 and 4), including GCFs of highly similar RiPPs, NRPSs, PKS-NRPS hybrids, and aryl-polyenes, without any homolog BGC described for the production of known metabolites (Fig. 3). Hence, we argue that SMs encoded by R. solanacearum plasmids may play relevant ecological roles and deserve further research to gain broader knowledge about the pathogenic mechanisms of this species.

Beyond plant-associated microbes, we also found SMs encoded in plasmids from relevant human-hosted bacteria, including pathogens. For instance, there is a very large GCC of siderophores within Klebsiella, *Cronobacter*, and Escherichia plasmids. These BGCs share high similarity with those producing aerobactin from *Pantoea* and *Xenorhabdus* isolates. This molecule can act as a virulence factor that contributes to extracellular pathogenesis and colonization of host tissues (46, 47). Hence, its plasmidic dispersion among members of *Enterobacteriaceae* can impact the human microbiome by enhancing virulent phenotypes. Similarly, we found several RiPPs clustered in a large clan of *Enterobacteriaceae* plasmids and two others hosted by Staphylococcus aureus strains. These cryptic BGCs are not related to known molecules, but considering the diverse biological activities of RiPPs (48, 49), the plasmidic dissemination of these BGCs may also have a deep impact on the human microbiome.

Despite the large diversity of BGCs found in the plasmid data set, based on our data, only 11.2% of the analyzed plasmids could produce an SM (see File S2 in the supplemental material). Indeed, as described above, this biosynthetic potential is mainly limited to a few host taxa. Interestingly, it seems that some plasmids merely contain the genetic material to produce SMs. While the occurrence of BGCs in plasmids is not common in microbes, it is the rule for some taxa. Therefore, we suggest that future ecological and evolutionary research on these microbes (i.e., rhizobia, R. solanacearum, *Bacillus*, *Streptomyces*) should include efforts to study all replicons and not be limited to chromosomal sequences (50, 51).

### Conclusions.

In summary, we show that some prokaryotes harbor nonnegligible biosynthetic potential within their plasmids. Nonetheless, this is not a common trend in microbes but only in a select group of host prokaryotes. We uncovered many different exclusive BGCs in clusters of analogous plasmids (PTUs) from plant- and human-related bacteria, suggesting that this genetic material may play relevant roles in host-associated microbiomes. Hence, our data provide a greater understanding of the functionality of microbial plasmids and the dynamics of SMs in prokaryotes. Finally, considering the availability of BGCs in wild plasmids and the ease of using these replicons to search for metabolites in laboratory conditions, we suggest that our findings can be applied to natural product research by prioritizing clans of BGCs with no homologs in public BGC databases.

## MATERIALS AND METHODS

### Obtaining plasmid sequences.

All available assembled plasmids in the RefSeq database were downloaded (https://ftp.ncbi.nlm.nih.gov/refseq/release/plasmid/). The bulk sequences were split into single files (one per plasmid) with the “split-fasta” command (https://github.com/uditvashisht/split-fasta), and each file was then renamed with the NCBI accession number. Only those classified as trusted plasmids of high quality by Redondo-Salvo et al. (17), which were used as input for the PTU classification (17), were retained. Then, we removed those with a total size of <1,000 nucleotides (nt) and those with gene accession codes (NG_*). In total, 9,183 plasmids were used for further analyses. More details on coding are provided in the GitHub repository for this article (https://github.com/zakisaati/plasmid_mining).

### Plasmid annotation and search for BGCs.

Gene calling and annotation of the plasmids was performed using Prokka v1.14.6 (52). Then, the GenBank annotated files were used as the input for annotation using antiSMASH (Antibiotics and Secondary Metabolite Analysis Shell) v6.1.1 (53), which searches for BGCs related to the biosynthesis of secondary metabolites. For further details on coding, see the supplementary methods within the GitHub repository for this article (https://github.com/zakisaati/plasmid_mining).

### Analysis of the diversity of BGCs across all plasmids.

We used BiG-SCAPE (Biosynthetic Gene Similarity Clustering and Prospecting Engine) v1.1.15 (15) to explore the diversity of all the annotated BGCs. This program allowed us to create sequence similarity networks (SSNs) among closely related BGCs and to classify the BGCs within gene cluster families (GCFs). We used the annotated BGCs in GenBank format as the input and activated “mix” mode to create a global SSN with every type of BGC. The gene cluster clan (GCC) setting was set as follows: the first cutoff parameter (network nodes are represented by the GCFs defined at this cutoff level) was 0.3; the cutoff for clustering families into clans was set to 0.7. We activated the search for similar BGCs within the Minimum Information about a Biosynthetic Gene Cluster (MIBiG) v3.1 database (54). The output SSN was rerendered using Cytoscape v3.7.2 (55). The phylogenetic trees of GCFs were constructed as described in the BiG-SCAPE/CORASON workflow (15), which uses a maximum likelihood approach over the core regions of homologous loci.

Graphics (scatterplots, bubble plots, and box plots) for comparison of the BGC content across host phyla and BGC types were created using ggplot2 (56) in R. For further details, see the supplementary methods on GitHub (https://github.com/zakisaati/plasmid_mining). Linear regression statistics were calculated using the “lm” function in ggplot2 (56).

### Data availability.

The source data and bioinformatic codes used in this work can be accessed in the GitHub repository (https://github.com/zakisaati/plasmid_mining). Plasmid sequences, BGC predictions, and networks can be accessed at Zenodo (https://doi.org/10.5281/zenodo.7816421).
