# Supplementary figures and images for: Global Map of Specialized Metabolites Encoded in Prokaryotic Plasmids
Source: Microbiol Spectr. 2023 Jun 13;11(4):e01523-23. doi: 10.1128/spectrum.01523-23 (PMC10434180; doi:10.1128/spectrum.01523-23)

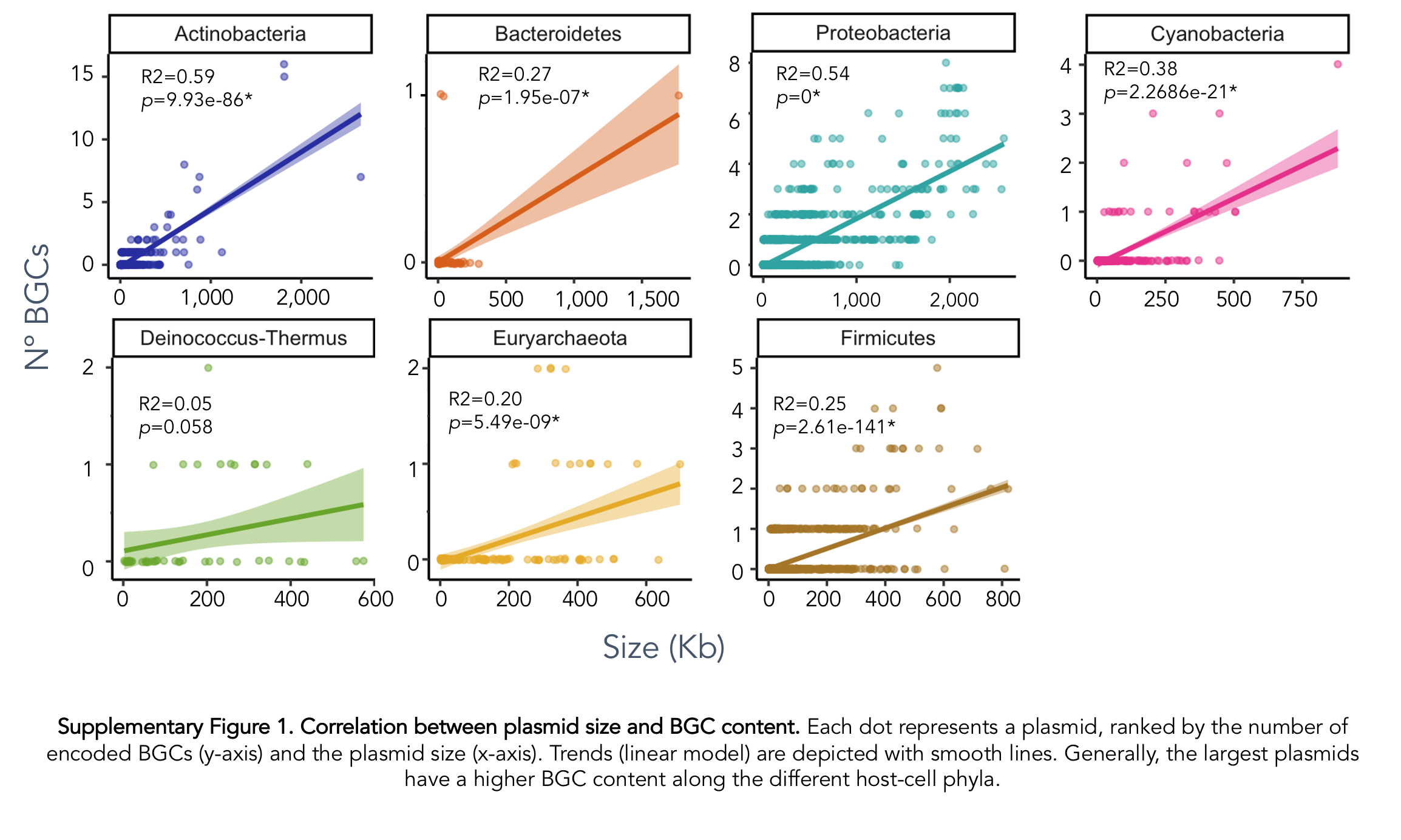

Supplement: Supplemental File 3 — File S3. Download spectrum.01523-23-s0003, PNG file, 0.5 MB [file spectrum.01523-23-s0003.png]
